# Understanding Waterfall Plots

**Published:** 2012-03-01

**Authors:** Theresa W. Gillespie

**Affiliations:** From Department of Surgery, Emory University School of Medicine, and Atlanta Veterans Affairs Medical Center, Atlanta, Georgia

Clinical trials in oncology continue to offer hope for tumor control. Such hope intensifies as the rapidly advancing knowledge from molecular biology, genetics, and immunology is more readily applied to targeted therapies. The ability to discern genomic or genetic risks and likelihood of response on a biologic basis has enhanced the field’s capacity to tailor therapeutic interventions specific to the individual’s tumor biology and possible host response. The impact of the tumor’s characteristics on outcome, as well as the effect of the patient’s characteristics, e.g. smoking status, become increasingly important to measure and interpret as these variables relate to response, progression-free survival (PFS), and ultimately, overall survival (OS).

For decades, overall survival has been the "gold standard" for interpreting clinical trials, as often results of PFS have not translated into long-term survival. In a similar way, Kaplan-Meier curves have long been used to illustrate survival analyses for clinical trials and other statistical evaluations of survival over time.

More recently, different types of graphs called *waterfall plots* have begun to gain in popularity in serving as the means to visually depict results of response to, and types of survival from, oncology clinical trials. An example of a recent publication that relied on waterfall plots for data presentation is Kwak et al.’s *New England Journal of Medicine* article entitled, "Anaplastic lymphoma kinase inhibition in non-small-cell lung cancer" (Kwak et al., 2010), discussed on page 103 by Beth Eaby-Sandy, MSN, CRNP, OCN®. Since these graphic presentations may be unfamiliar to some readers, this article will comment on their interpretation and application herein.

## What Do Waterfall Plots Illustrate?

Waterfall plots are graphic illustrations of data that can vary from audio frequencies to clinical trial patient information and results. In oncology, for example, a waterfall plot may be used to present each individual patient’s response to a particular drug based on a parameter, such as tumor burden. The horizontal (x) axis across the plot may serve as a baseline measure; vertical bars are drawn for each patient, either above or below the baseline. The vertical (y) axis may be used to measure maximum percent change from baseline, e.g., percent growth or reduction of the tumor by radiologic measurement. Those vertical bars that are above the line represent nonresponders or progressive disease. Vertical bars below the baseline (x) axis are drawn for each patient that has achieved some degree of tumor reduction, often depicted as negative percent.

**Figure 1:**
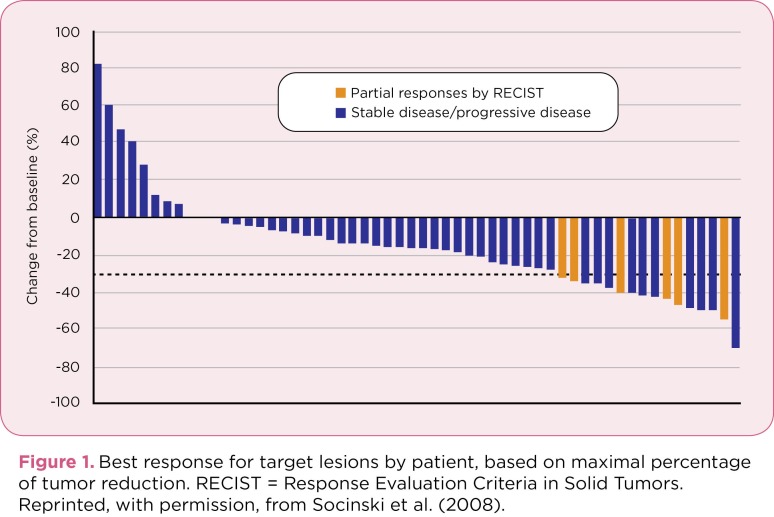
Figure 1. Best response for target lesions by patient, based on maximal percentage of tumor reduction. RECIST = Response Evaluation Criteria in Solid Tumors. Reprinted, with permission, from Socinski et al. (2008).

In general, waterfall plots go from the worst value, such as greatest progression of disease, on the left side of the plot, to the best value, i.e., most reduction of tumor, on the right side of the plot (Socinski et al, 2008); this can also be shown by shifting the graph to a similar presentation, moving from the worst outcomes on the bottom to the best outcomes on the top. The length of each vertical bar hanging below the horizontal axis increases as the plot moves to the right side of the graph, thus resembling a waterfall and giving the graph its name. Thus, the data are not presented randomly, or in order of when a patient first enrolled in a trial, but are organized in order to provide a clear picture of the study population’s results: from worst to best, based on the parameters included. An example of a waterfall plot is shown in Figure 1 (Socinski et al., 2008).

The individual bars, besides representing a single subject, can also be used to represent other key patient characteristics using a different color or pattern, such as the type of response achieved by the subject (e.g., stable vs. partial), or whether the subject was a smoker or nonsmoker. Consequently, the waterfall plot provides data about not only a primary outcome, such as objective response, but also additional relevant information about *what type of patient* is achieving that outcome—for example, nonsmokers.

As shown in Figure 1 (Socinski et al., 2008), from a study of sunitinib (Sutent), many patients experienced tumor shrinkage that did not meet the response criteria for objective response. When these patients are presented as part of a waterfall plot, as one reads the graph moving from left to right, the findings show considerable antitumor activity from which patients may benefit, even if the patient evaluations do not result in documented partial or complete response by RECIST (Response Evaluation Criteria in Solid Tumors). Thus, having a visual presentation with a waterfall plot may enhance understanding of response data, particularly for stable disease.

## Waterfall Plots in the Kwak et al. Article

In the article by Kwak et al. (2010), a population of patients with non–small cell lung cancer, whose tumors were positive for anaplastic lymphoma kinase (ALK) rearrangements (N = 82), were enrolled on a clinical trial of crizotinib (Xalkori), an oral inhibitor of ALK and other kinases that inhibit tyrosine phosphorylation of activated ALK. Imaging assessments of tumor response were performed using RECIST v1.0 (Wahl, Jacene, Kasamon, & Lodge, 2009). Progression-free survival was calculated from the date of the first crizotinib administration to the date when objective progression of disease was documented or the date of death from any cause. Kaplan-Meier (KM) methods were used to examine PFS and OS. All analyses used two-sided confidence intervals and SAS statistical software v9.2 (Allison, 1995). In this article, waterfall plots were used to represent a variety of results.

**Figure 2:**
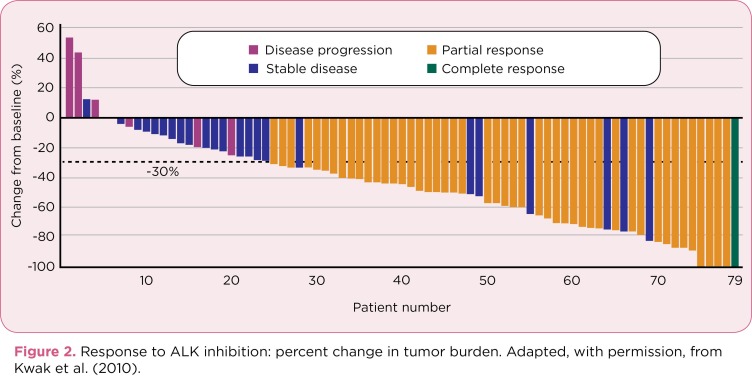
Figure 2. Response to ALK inhibition: percent change in tumor burden. Adapted, with permission, from Kwak et al. (2010).

In Figure 2, percent change in tumor burden is illustrated. In addition, the type of response is displayed using different colors. Thus, the reader can quickly determine both the number of patients who experienced a response of any type, as well as the distribution of stable, partial, and complete responses for the group.

**Figure 3:**
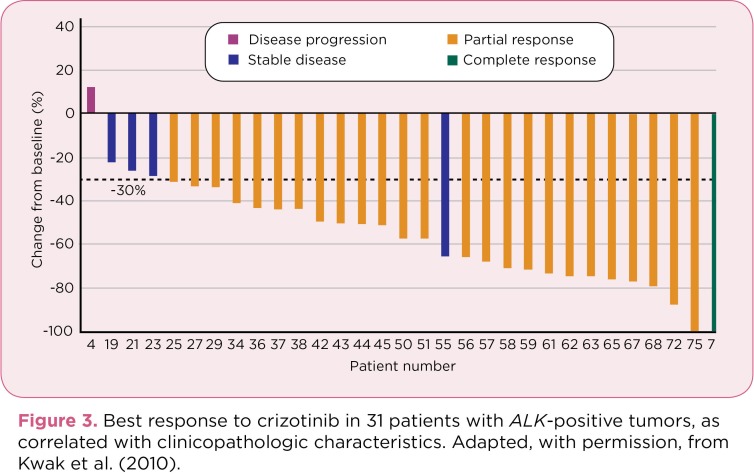
Figure 3. Best response to crizotinib in 31 patients with ALK-positive tumors, as correlated with clinicopathologic characteristics. Adapted, with permission, from Kwak et al. (2010).

**Figure 4:**
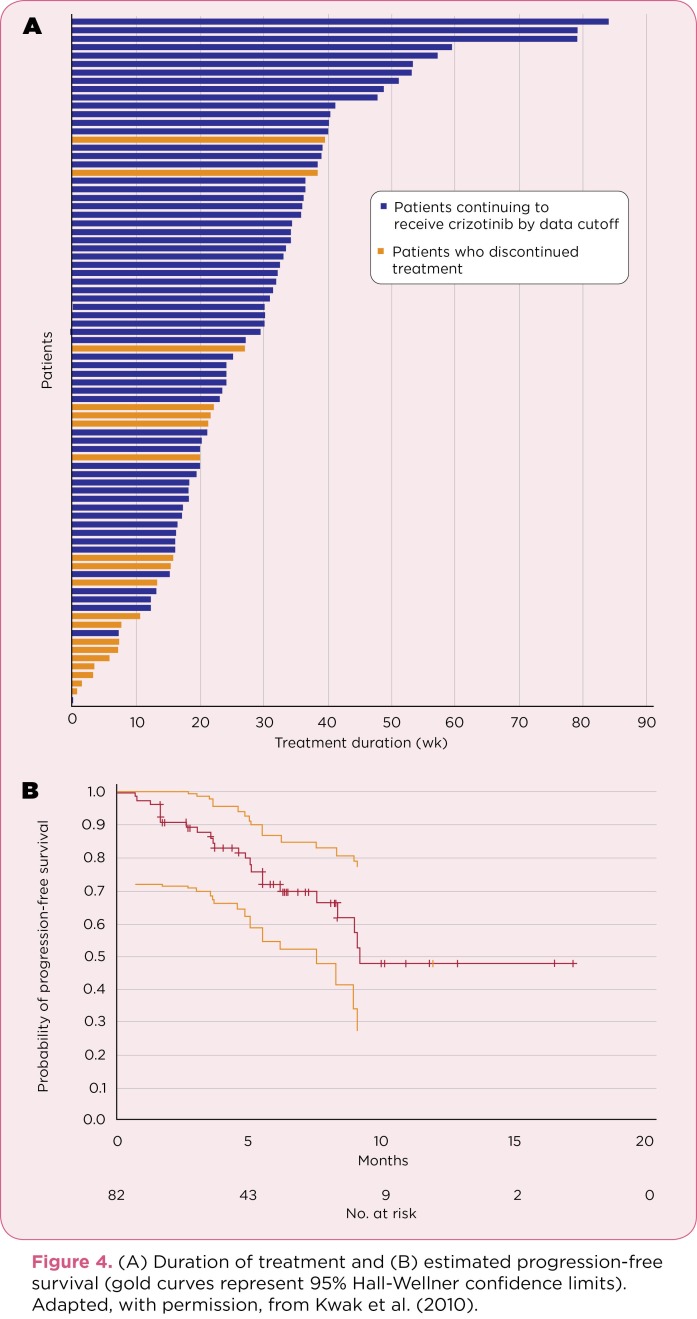
Figure 4. (A) Duration of treatment and (B) estimated progression-free survival (gold curves represent 95% Hall-Wellner confidence limits). Adapted, with permission, from Kwak et al. (2010).

In Figure 3, the authors display best response to crizotinib with clinicopathologic correlation. Additional data related to each patient that might contribute to the response status are included in the figure text. An additional graph (Figure 4) compares duration of treatment and KM curves and is explained below.

## How Do Waterfall Plots Differ From Other Analytic Graphs?

Kaplan-Meier estimates, or survival curves, are perhaps the most widely used method for estimating survival in biomedical analyses (Wahl et al., 2009). Many oncology clinicians and researchers are accustomed to seeing and interpreting KM outputs of OS in which a symbol, e.g., a dot on the graph, represents each subject; as each death occurs, the curve drops down to a lower value, illustrating the percent of the sample still surviving at that time. Kaplan-Meier estimations become more complex depending on whether and at what time interval censoring takes place with the sample being observed. Those subjects in the study population who have died will be represented by declines in the survival curve, while those subjects who remain alive will continue on the line across the x axis, creating a proportion (e.g., 30%) of subjects still alive at a given cutoff date when the data were analyzed. A KM estimate can also be produced for disease-free survival or other estimates.

The differences between a waterfall plot and a KM estimate are shown in the Kwak et al. article; see Figure 4, panels A and B, respectively. Duration of treatment in a waterfall plot is depicted in Figure 4, panel A. All 82 patients are included as represented by the horizontal bars, one for each subject. Nineteen patients are shown in gold, indicating 19 subjects who had discontinued treatment at the time of the cutoff date, with reasons given for discontinuation listed in the figure text. In this particular waterfall plot, the increasing length of treatment duration is illustrated from the bottom (least treatment duration) to the top (longest duration) of the graph, rather than increasing from left to right as shown on the earlier plots (Figures 2 and 3) in the same article.

In Figure 4, panel A, it appears that 11 patients were continuing to take crizotinib at 40 weeks, which is similar to about 9 patients shown on the Kaplan-Meier PFS estimate for 10 months in panel B. In panel A, one patient is shown as continuing treatment beyond week 80, with all other patients shown as stopping prior to that point. In panel B, all 82 patients are represented in a KM estimate of PFS. Each patient who has died creates a drop in the KM curve, with an estimate of 50% probability of PFS occurring at about 10 months. The number of subjects at risk range from the 82 who started to 9 at the 10-month point, with a probability of 2 subjects estimated for the 15-month point.

One can compare KM curves to waterfall plots on several different elements, including ease of visualization, detail of data represented, and speed of interpretation. But just as shown by Kwak et al., it may be beneficial to combine different graphic presentations of data to illustrate varied statistical findings. As waterfall plots become more common in oncology practice and clinical trials, familiarity with such statistical presentations will increase and the reader’s level of comfort with accurately interpreting such graphs will also likely expand.

## Using Waterfall Plots to Illustrate Oncology Data: Advantages and Disadvantages

Like any other technique or statistical method, waterfall plots have both advantages and disadvantages. One advantage is that waterfall plots can serve as a novel efficacy measure, for both cytotoxic agents and biologic or cytostatic agents, in terms of presenting the reduction in tumor burden for each subject (Campbell, Grothey, Sargent, & Goldberg, 2007). A second advantage may relate to interpretation of stable disease as graded with RECIST criteria (Campbell et al., 2007). Kwak et al. reported results that showed impressive objective responses, but stable disease is a frequent finding in many trials and not easily interpreted in terms of clinical benefit. Stable disease can range from a small amount of tumor growth to a modest amount of tumor reduction, but insufficient change in the tumor to be evaluated as an objective response to therapy or as progressive disease.

While waterfall plots may be excellent tools to present the variability of clinical findings, disadvantages or limitations of their application exist. Since the vertical plots in the waterfall graph each represent an individual patient along a horizontal line, waterfall plots work best with a smaller cohort of patients. For larger populations, the waterfall plot presentation can become unwieldy as the graph may need to stretch beyond optimal visualization in order to accommodate a larger patient group. In addition, a study using a randomization scheme other than 1:1 will not lend itself as well to a waterfall plot technique. As stated previously, since each vertical plot represents a single patient, waterfall plots limit the ability to portray different randomization schemes, e.g., 2:1 or 3:1, in a manner that is readily illustrated and clearly comprehended.

## Summary

Like any statistical or clinical method, waterfall plots illustrate information about a treatment that works well for certain patients but not as well for others. As data become clearer about why certain patients may or may not respond to specific treatments, looking at those patients on both the far right and the far left of the waterfall plot presentation becomes increasingly important. In the quest to attempt personalization of treatment for individual patients, the waterfall plot can facilitate clinical treatment decision-making. Determining the basis for the more responsive or longer durations of response demonstrated by the subjects on the right, as well as the underlying reasons for the lack of such response in the individuals depicted in the bars shown on the left (or bottom) of a waterfall plot, may help focus future research and design of clinical trials. In the meantime, waterfall plots serve as a useful tool in the armamentarium of visual and statistical methods available to present and interpret findings from oncology clinical trials and other research pursuits.
